# External Stresses Affect Gonococcal Type 4 Pilus Dynamics

**DOI:** 10.3389/fmicb.2022.839711

**Published:** 2022-02-25

**Authors:** Sebastian Kraus-Römer, Isabelle Wielert, Isabel Rathmann, Jan Grossbach, Berenike Maier

**Affiliations:** ^1^Institute for Biological Physics, University of Cologne, Cologne, Germany; ^2^Faculty of Mathematics and Natural Sciences, CECAD, University of Cologne, Cologne, Germany; ^3^Center for Molecular Medicine Cologne, Cologne, Germany

**Keywords:** type 4 pilus, antibiotics, reactive oxygen species, molecular motor, *Neisseria*, transcriptome

## Abstract

Bacterial type 4 pili (T4P) are extracellular polymers that serve both as adhesins and molecular motors. Functionally, they are involved in adhesion, colony formation, twitching motility, and horizontal gene transfer. T4P of the human pathogen *Neisseria gonorrhoeae* have been shown to enhance survivability under treatment with antibiotics or hydrogen peroxide. However, little is known about the effect of external stresses on T4P production and motor properties. Here, we address this question by directly visualizing gonococcal T4P dynamics. We show that in the absence of stress gonococci produce T4P at a remarkably high rate of ∼200 T4P min^–1^. T4P retraction succeeds elongation without detectable time delay. Treatment with azithromycin or ceftriaxone reduces the T4P production rate. RNA sequencing results suggest that reduced piliation is caused by combined downregulation of the complexes required for T4P extrusion from the cell envelope and cellular energy depletion. Various other stresses including inhibitors of cell wall synthesis and DNA replication, as well as hydrogen peroxide and lactic acid, inhibit T4P production. Moreover, hydrogen peroxide and acidic pH strongly affect pilus length and motor function. In summary, we show that gonococcal T4P are highly dynamic and diverse external stresses reduce piliation despite the protective effect of T4P against some of these stresses.

## Introduction

Type 4 pili (T4P) are polymeric cell appendages that are responsible for a remarkable variety of functions in the bacterial world. These functions include adhesion to host cells and other surfaces, microcolony formation, twitching motility, horizontal gene transfer, and surface sensing (Berry and Pelicic, 2015; Craig et al., 2019). In contrast to most other extracellular structures, their length is highly dynamic and T4P retraction generates mechanical force (Merz et al., 2000; Skerker and Berg, 2001; Maier et al., 2002). Dynamics and force generation are crucial for many T4P associated functions. Therefore, it is important to quantify T4P dynamics in different environmental conditions.

Bacterial aggregation causes tolerance against a large number of antibiotics (Hall and Mah, 2017). The potential mechanisms include hindered diffusion of the drug within the colony (Tseng et al., 2013; Singh et al., 2016), stress responses (Nguyen et al., 2011; Secor et al., 2018), and community-related differentiation (Yan and Bassler, 2019). In many bacterial species, aggregation is controlled by T4P and therefore, piliation and T4P dynamics likely affect stress tolerance. For example, piliation increased the tolerance against the β-lactam antibiotic ceftriaxone (Stohl et al., 2013) and hydrogen peroxide (Wang et al., 2018) in the human pathogen *N. gonorrhoeae*. Susceptibility for this drug can be modulated by fine-tuning T4P-T4P interactions between neighboring cells within the colonies. In particular, posttranslational modification of the major pilin subunit PilE or variation of the activity of the T4P retraction motor PilT affect the strength of cell-to-cell attachment (Bonazzi et al., 2018; Welker et al., 2018). We have shown recently, that even small changes in T4P-T4P interaction cause order of magnitude changes in the viscosity of the bacterial colonies (Zollner et al., 2019; Maier, 2021). This change in viscosity correlates with bacterial survival under antibiotic treatment (Cronenberg et al., 2021); when the fluidity is increased, the survival rate under ceftriaxone treatment decays. Importantly, antibiotic treatment affects colony fluidity (Cronenberg et al., 2021). In particular, the macrolide azithromycin and the fluoroquinolone ciprofloxacin strongly enhance colony fluidity, suggesting that T4P dynamics may be altered by the action of the antibiotics.

The T4P fiber is composed of major and minor pilins which are stored in the inner membrane of bacteria. The fiber is extruded through a T4P complex that spans the inner membrane, the periplasm, and the outer membrane (Craig et al., 2019). This complex is stable in the absence of the T4P fiber (Friedrich et al., 2014; Chang et al., 2016). At the cytoplasmic site of the T4P complex either an elongation ATPase (PilF) or a retraction ATPase (PilT) bind, supporting T4P elongation or retraction, respectively (Freitag et al., 1995; Wolfgang et al., 1998). T4P dynamics have been studied by microscopic techniques involving laser tweezers (Merz et al., 2000; Clausen et al., 2009; Ribbe et al., 2017), micropillars (Biais et al., 2008), and fluorescence microscopy (Skerker and Berg, 2001; Koch et al., 2021). Laser tweezers and micropillars probe mechanical effects of T4P retraction and enable measurement of T4P generated force. Laser tweezers with force feedback allow for characterization of T4P retraction speed at high accuracy (Clausen et al., 2009) but they are insensitive to T4P elongation. Gonococcal T4P were shown to retract at a speed of 2 or 1 μm/s, respectively, depending on oxygen availability (Kurre and Maier, 2012). Early protocols for fluorescence imaging (Skerker and Berg, 2001; Eriksson et al., 2015) were recently improved using maleimide chemistry (Ellison et al., 2019) and proven useful for characterizing T4P elongation and retraction in various bacterial species (Ellison et al., 2017, 2018; Lam et al., 2021; Vesel and Blokesch, 2021). In particular, the fluorescence method was used for quantifying T4P dynamics in detail in *Pseudomonas aeruginosa* (Koch et al., 2021). The results strongly support a model of stochastic and competitive binding between the extension ATPase PilF and the retraction ATPase PilT in excellent agreement with cryo-electron microscopy studies (Koch et al., 2021) and laser tweezers studies (Clausen et al., 2009). In these rod-shaped *P. aeruginosa*, T4P were mainly formed at the active pole, where most of the membrane-standing T4P complexes resided (Koch et al., 2021).

Here, we employ the fluorescence microscopy technique for investigating the dynamics of all T4P in spherically shaped and peritrichously piliated *N. gonorrhoeae* and develop image analysis tools for characterizing T4P dynamics. At the level of the entire cell, we characterize the rate of T4P production and T4P density. At the level of single T4P, we measure the velocities of T4P elongation and retraction, the maximum length that a T4P reaches prior to the onset of retraction, and the T4P lifetime. Combining these tools with RNA sequencing, the effects of the two antibiotics whose application is currently recommended against gonorrhea, azithromycin and ceftriaxone (Prevention CfDCa, 2015), are investigated. We present evidence that application of both drugs reduces piliation by downregulating the membrane-standing T4P complex that extrudes the T4P fiber. Furthermore, we investigate effects of antibiotics affecting various targets, and of natural stressors of *N. gonorrhoeae*. By combining all data, we find evidence that external stresses reduce the rate of T4P production by reducing the density of T4P complexes. Since piliation has been shown to protect gonococci from diverse external stresses, the reduction of piliation is likely to amplify the damage caused by these stresses.

## Materials and Methods

### Bacterial Strains and Growth Media

*Neisseria gonorrhoeae* was grown overnight at 37°C and 5% CO_2_ on agar plates containing gonococcal base (GC) agar [10 g/l Bacto agar (BD Biosciences, Bedford, MA, United States), 5 g/l NaCl (Roth, Darmstadt, Germany), 4 g/l K_2_HPO_4_ (Roth), 1 g/l KH_2_PO_4_ (Roth), 15 g/l Proteose Peptone No. 3 (BD), 0.5 g/l soluble starch (Sigma-Aldrich, St. Louis, MO, United States)] supplemented with IsoVitaleX (IVX): 1 g/l D-Glucose (Roth), 0.1 g/l L-glutamine (Roth), 0.289 g/l L-cysteine-HCL × H_2_0 (Roth), 1 mg/l thiamine pyrophosphate (Sigma-Aldrich), 0.2 mg/l Fe(NO_3_)_3_ (Sigma-Aldrich), 0.03 mg/l thiamine HCl (Roth), 0.13 mg/l 4-aminobenzoic acid (Sigma-Aldrich), 2.5 mg/l β-nicotinamide adenine dinucleotide (Roth) and 0.1 mg/l vitamin B_12_ (Sigma-Aldrich). GC medium is identical to the base agar composition but lacks agar and starch.

*E. coli* was grown in LB (Lysogeny Broth, Roth) medium or on LB agar plates (15 g/l Bacto agar (BD Biosciences, Bedford, MA, United States) at 37°C.

For *N. gonorrhoeae* antibiotics were used at the following concentrations: 2.5–5 μg/ml erythromycin (Thermo-Fisher), 100 μg/ml streptomycin (Sigma-Aldrich), 10 μg/ml chloramphenicol (Sigma-Aldrich). For *E. coli* antibiotics were used at the following concentrations: 50 μg/mL kanamycin (Roth).

### Construction of Strain Carrying Cys-Modification Within Major Pilin

First, a fragment containing the promoter of the major pilin, *pilE*, and the *pilE* gene, *P_*pilE*_pilE*, were amplified from genomic DNA (ΔG4, Ng150, Supplementary Data Sheet 2) with primers sk5 and sk40 (Supplementary Data Sheet 3). The PCR product was digested with XhoI and AflII (New England Biolabs), as well as the *piga* vector. The digested products were ligated and subsequently transformed into *E. coli* DH5α. After selection on kanamycin, plasmid DNA of positive clones were isolated with the GenUP™ Plasmid Kit (biotechrabbit GmbH, Berlin, Germany) according to the manufacturer’s instructions.

Then, the threonine at position 126 was replaced by cysteine as follows. *Via* site-directed mutagenesis with the KAPA polymerase (Roche) and primer sk43 and sk44 threonine at position 126 was substituted by a cysteine resulting in *pilE^T126C^*. Afterward, the PCR reaction was digested with DpnI (New England Biolabs) to reduce the amount of template DNA. Subsequently, the digested PCR product was transformed into *E. coli*. Plasmid DNA was purified with the GenUP™ Plasmid Kit (biotechrabbit) according to the manufacturer’s instructions and sequenced with primers sk9 and sk10.

Subsequently, the native *pilE* sequence was replaced by *pilE^T126C^* with a “clean substitution.” To this end, we used a two-step selection process with a *ermC/rpsL* cassette as described in Dillard (2011). Δ*G4* (Ng150) was transformed with the fusion PCR product 5′UTRpilE- pilE*^T126C^*-ermC-rpsLs-3′UTRpilE and selected on erythromycin. Insertion was controlled *via* PCR with primers sk32 and sk45. 5′UTRpilE pilE*^T126C^* was amplified using primer sk129 and sk147 from plasmid DNA piga::*P_*pilE*_pilE*^T126C^**. Primer sk146 and sk135 were used to amplify the *ermC-rpsL_*s*_* construct. 3′UTR was amplified using primer sk143 and sk145. The PCR products were fused and then the PCR product was transformed into Δ*G4 (Ng150)* and selected on erythromycin resulting in strain Δ*G4 pilE^T126C^* step1 (Ng225). To check for correct insertion, the transformants were controlled *via* screening PCR. Subsequently, Δ*G4 pilE*^T126C^** step1 was transformed with the fusion PCR product 5′UTR pilE- pilE*^T126C^*- 3′UTRpilE and counter-selected on streptomycin. 5′UTR pilE- pilE*^T126C^* was amplified from Δ*G4 pilE*^T126C^** step1 (Ng225) with primer sk129 and sk131. 3′UTR was amplified from Δ*G4 (Ng150)* using primer sk132 and sk158. The PCR products were fused and transformed into Δ*G4 pilE*^T126C^**step1 (Ng225). During a successful “clean substitution” the *ermC* resistance gene and the dominant streptomycin-sensitivity allele *rpsL*_*s*_ are spliced out and the strain is again resistant to streptomycin. Insertion was controlled *via* PCR with primers sk32 and sk45 and subsequently checked *via* sequencing with primer sk129. After counter-selection, strain ΔG4 *pilE^T126C^* (Ng226) is isogenic to ΔG4 (Ng150) besides the amino acid exchange within *pilE.*

To construct strain Ng250, strain ΔG4 *pilE^T126C^* (Ng226) was transformed with genomic DNA containing *pilT::m-Tn3cm* (Aas et al., 2007) and selected on chloramphenicol.

### T4P Fluorescence Labeling

In order to visualize gonococcal T4P the protocol of Ellison et al. (2018) was adapted. Strain Δ*G4 pilE*^T126C^** (Ng226) was used for pilus labeling. The OD600 was adjusted to 0.1 in 100 μL cysteine-free retraction assay medium (RAM) consisting of phenol red-free Dulbecco’s Modified Eagle Medium (GIBCO, Grand Island, NY, United States), 4.5 g/l Glucose (GIBCO), 2 mM L-glutamine (Roth), 8 mM sodium pyruvate (GIBCO), and 30 mM HEPES (Roth). Subsequently, 62 μM AF488 Alexa Fluor 488 C5 Maleimide (Thermo-Fisher) was added and cells were incubated for approximately 1 h at 37°C and 250 rpm. Then cells were washed and resuspended in GC media. A droplet of the resuspended cells was transferred onto a glass slide attached to a sticky-Slide 8 well (ibidi GmBH, Germany). A GC agar pad was put on top of the droplet and cells were imaged after a short incubation time (37°C and 5% CO_2_).

To investigate the effect of antibiotic treatment on T4P characteristics, we incubated the cells before (2h) and during labeling (1 h) with antibiotics at twofold MIC with the following antibiotics: 0.256 μg/ml azithromycin (hello bio, Dunshaughlin, Ireland), 0.016 μg/ml ceftriaxone (hello bio.), 0.008 μg/ml ciprofloxacin (Sigma-Aldrich) and 0.128 μg/ml streptonigrin (Sigma-Aldrich). Controls were treated with the same volume of either DMSO (Roth) or 0.1 M HCl.

Oxidative Stress was applied in form of hydrogen peroxide (Roth). Cells were treated after labeling for 15 min with 1 mM H_2_0_2_, 2 mM H_2_0_2_ or the same volume of water as control.

Furthermore, the effects of pH on T4P characteristics were analyzed. GC medium was supplemented with 100 mM lactic acid (Roth) and adjusted to a pH of 5.5, 6.3, 7.0, and 7.7 with 10 M NaOH (Roth). During the labeling process, the pH of RAM was adjusted to the respective pH. Subsequently, cells were incubated for 10 min in pH-adjusted GC media prior to imaging.

### Confocal Microscopy

Images were acquired using an inverted microscope (Ti-E, Nikon) equipped with a spinning disc confocal unit (CSU-X1, Yokogawa) and a 100×, 1.49 NA, oil immersion objective lens. The excitation wavelength was 488 nm. For pilus dynamic analysis, movies were generally recorded for 20 s with an auto exposure of 50 ms, resulting in a frame-rate of 19.33 frames/s. We analyzed only individual and well separated gonococci.

### Determination the Rate of T4P Production and Number of T4P

All image processing steps and the analysis explained in this section were performed with MATLAB 2021. A region of interest of 50 × 50 pixels was defined for one bacterium per recorded video. Only bacteria, that show dynamic T4P were analyzed and, therefore, dead or inactive cells are excluded from the analysis. The image of a single T4P is diffraction-limited and, therefore, has a diameter of 3 Px (0.25 μm). The labeling of the cell envelope poses problems with T4P analysis. Supplementary Figure 1 shows that the fluorescence intensity of a single T4P is considerably weaker compared to the intensity of the contour. Thus, the contrast was adjusted and filters were applied to reduce the noise as explained in the following.

We determined the average number of present pili, N_*p*_, as well as the T4P production rate, r_*p*_, as follows. A contour encircling the cell body was generated following the shape of each bacterium including monococci and diplococci. First, two binary masks of the cell body were created by setting a threshold above the intensity of the T4P. One of the masks was then enlarged by creating a disk-shaped structuring element from the center of mass of the original binary mask of the cell. The magnification of this mask depends on the average radii of the bacteria for each condition, therefore the radius of the disk-shaped structuring element was adapted to the condition we analyzed (control, pH, H_2_O_2_: 9 Px; azithromycin treatment: 7 Px; ceftriaxone treatment: 13 Px). For the magnification the MATLAB function *imdilate* was used and the *strel* function was used to create the structuring element. Subsequently, the original mask was subtracted from the magnified mask, resulting in a contour with a width from 1 to 2 pixels (Supplementary Figure 2A).

If a new T4P elongates from the cell body and crosses the contour, the fluorescence intensity at the respective position along this contour increases step-wise. When a T4P is fully retracted, the fluorescence intensity decreases step-wise to the background level. To detect the crossing of the pili through the contour, the contour of the cell was subdivided in 80 sectors emanating from the center of mass of the cell body (Supplementary Figure 2A). Each sector represents an angular division of 4.5°. Then, for each frame (time point) the contour was projected onto a line by averaging over all pixels of the contour which were assigned to the same sector. A wiener filter *wiener2* (MATLAB) was applied to the kymograph. To reduce the noise and enhance filamentous structures, the *fibermetric* function of MATLAB [*fibermetric* enhances filamentous objects *via* a Hessian based multiscale filter (MATLAB)] was applied (Supplementary Figure 2B). Next, the peaks corresponding to T4P were detected *via* the *peak* function (MATLAB) for each frame along the sectors of the contour (Supplementary Figure 2C). To avoid that noise is detected as a T4P or cuts a single T4P into two T4P, peaks that lasted for less than 0.5 s were disregarded and two detection events were connected if the pausing in detection in one sector was below 0.5 s. Sometimes, T4P fluctuate between two sectors. Therefore, peaks detected in neighboring sectors were connected. The rate of pili production, r_*p*_, is rate number of newly generated T4P per cell and unit time. The occurrence of a new T4P was detected as a stepwise increase in fluorescence intensity at the contour around the cell. For example, in Supplementary Figure 2B new T4P occur around sector 10 at 2, 3, 5, and 7 s. To obtain r_*p*_, the number new T4P detected around the contour are summed over 7.5 s and divided by 7.5 s. To obtain the average rate of T4P production, all data were averaged over multiple bacteria. The number of T4P present at a specific time point, N_*p*_, is determined as the number of peaks along the contour (Supplementary Figure 2C). To obtain the mean number of T4P, the data were averaged over 7.5 s for each cell, and over multiple cells. All data were acquired on at least 3 days.

There are several limitations to the methods described above. First, short T4P with a length shorter than ∼ 0.8 μm are not detectable. Please note that this minimum length is shorter for other types of analyses, e.g., the T4P length distribution or the dynamics of individual T4P. Second, the number of detectable T4P is strongly reduced while bacteria are illuminated with laser light (Supplementary Figure 3). This effect is likely to due to photobleaching. Additionally, illumination of the fluorescent dye is likely to stress the bacteria by production of radicals and thus reduce the rate of T4P production. For this reason, we analyzed only the initial 7.5 s.

### Analysis of Single T4P Dynamics

The analysis of T4P dynamics was performed using MATLAB 2021. First, we chose a region of interest around the cell. Then, we applied a two-dimensional median filter to reduce noise. The last step was to apply a wiener filter *wiener2* (MATLAB) filter to reduce the noise which was amplified by adjusting the contrast. Subsequently, we chose the pili of the cell which were suitable to characterize and analyze. The criteria for analyzing the dynamics were as follows: (a) Pili point straight and radially away from the cell surface. (b) The pilus is clearly distinguishable from the noise of the cell body, which means, in general longer than about 0.7 μm.

In the next step, we rotated the video such that the pilus is directed in horizontal direction and a rectangular region of interest around one pilus was chosen (Supplementary Figure 4A). Then, a second region of interest with a width of five pixels was chosen as shown in Supplementary Figure 4B. The intensity profile within the region was averaged vertically. This procedure was repeated for every frame. To create the kymograph, all intensity profiles were plotted next to each other. The goal of the kymograph was to determine the elongation and retraction velocities. We edited the kymograph first by adjusting the contrast and then we detected the edges (Supplementary Figure 4C) which provided us with the track of the positions of the pilus tip. To obtain the velocity of T4P elongation and retraction, we determined the slope between the start and the end of each event (Supplementary Figure 4D). The procedure was repeated for each T4P of a gonococcus.

### Analysis of T4P Length

We measured the maximum length of T4P, T_p_, i.e., the length of each T4P at the time point where the pilus switched from elongation to retraction. All steps were performed with ImageJ (Fiji). We cropped a region of interest around a single gonococcus. Next, we summed all intensity values for the first hundred frames. Then, the contrast was adjusted and the image was sharpened (Supplementary Figure 5). From this image, we measured the length of every detectable pilus. As the noise of the cell body is still prominent, we decided that pili below a length of 0.5 μm were below the resolution of the measurement.

### RNA Isolation, Sequencing, and Transcriptome Analysis

Strain Δ*G4* (Ng150) was grown in a 24 well plate (Greiner Bio-One) in 1 mL cultures inoculated with approximately 3 × 10^6^ cells. Cells were grown for 6h at 37°C, 5% C0_2_ in an Infinite M200 plate reader with a shaking period of 2 min per OD cycle (OD was measured every 10 min). After 6h growth, azithromycin or ceftriaxone were added at twofold MIC or an equal amount of DMSO as control. After 15 and 60 min samples were treated with RNA protect bacteria reagent (Qiagen, Hilden, Germany) according to the manufacturer’s instructions. Pellets were stored in −80°C until total RNA was isolated by using the Qiagen RNeasy Protect Bacteria Mini Kit (Hilden, Germany) according to the manufacturer’s instructions. RNA was isolated from each condition from three different days each representing one batch. RNA samples were sent to Cologne Genomic Center (Cologne, Germany) for next-generation sequencing (NGS) and depletion of ribosomal RNA. Sequencing was performed on an Illumina HiSeq system with 100 bp paired reads and on average 10 million reads per sample. The RIN values of each sample were ≥ 9.9.

The reads from each library were trimmed and paired with Trimmomatic (version 0.36) (Bolger et al., 2014). The reads were then mapped against the reference genome of Neisseria gonorrhoeae MS11 (NCBI, CP003909.1) using STAR (2.5.3a) (Dobin et al., 2013). For calling read counts of all known genes of the reference, we used the program featureCounts from the subread package (version: 2.0.1) (Liao et al., 2014). We calculated the differential gene expression with the DESeq2 package implemented in R, controlling for batch differences between days. For each gene we then obtain the log2-fold change of the antibiotic treatment with respect to the DMSO-treated control, as well as the associated adjusted *p*-value. A gene is regarded as differentially regulated with if the (adjusted) *p*-value is ≤ 0.05 and the | log2-fold change| ≥ 0.5. The genes required for assembling the T4P core complex were annotated manually, the data can be found in Supplementary Data Sheet 2.

Genes with a copy number > 1 were excluded from the analysis, leaving 1,960 genes. For each gene, the ortholog in *N. gonorrhoeae* FA1090 (NC_002946.2) strain was determined *via* blastn (Camacho et al., 2008). Functional enrichment was performed for 17 functional categories, that were based on KEGG ontology (McClure et al., 2020) with the one-sided Fisher’s exact test and Bonferroni correction. These categories were condensed such that each gene is associated with one category, as can be found in Supplementary Data Sheet 3.

## Results

### Gonococci Generate T4P at a Remarkably High Rate

**FIGURE 1:**
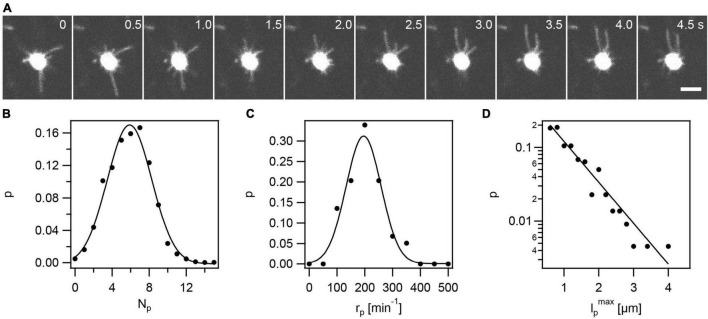
Visualization of fluorescent T4P in *N. gonorrhoeae* (Ng226). **(A)** Time lapse of gonococcus with AF488-labeled T4P. Scale bar: 2 μm. **(B)** Circles: Probability (p) distribution of number of T4P per cell, N_*p*_. Full line: Gaussian fit with ⟨*N*_*p*_⟩ = 5.9 ± 0.1 (mean from 59 cells ± standard error). **(C)** Circles: Probability distribution of rates of T4P production per cell, r_*p*_. Full line: Gaussian fit with ⟨*r*_*p*_⟩ = (196 ± 3)*min*^−1^ (mean of 59 cells ± standard error). **(D)** Circles: Probability distribution of T4P lengths l_*p*_*^max^* (derived from 220 T4P). l_*p*_*^max^* specifies the maximum length of a T4P, i.e., the length at the point in time where the T4P switches from elongation to retraction. Full line: exponential fit with characteristic length $l_{p}^{c}=\left(0.78\pm 0.06\right)\,\mu \,m$.

In the first step, we characterized gonococcal T4P dynamics in the absence of external stress. To this end, we adapted a T4P labeling protocol that was previously developed for *Caulobacter crescentus* and *Vibrio cholerae* (Ellison et al., 2019). A cysteine was introduced into the hypervariable region of the major pilin PilE enabling modification with the fluorescent dye Alexa Flour488 C_5_ maleimide as described in the Methods. Fluorescently labeled gonococci were sandwiched between a cover glass and an agar pad and imaged using confocal microscopy. Wt gonococci showed impressively dynamic T4P (Figure 1A and Supplementary Movie 1). Typically, multiple T4P were present simultaneously at different stages of elongation and retraction. As expected, deletion of the retraction ATPase PilT inhibited T4P dynamics (Supplementary Figure 6), confirming that the observed dynamics depends on PilT.

The gonococcal cell envelope shows a strong fluorescent signal (Figure 1A and Supplementary Figure 7). Application of the dye to wt gonococci that do not carry the cysteine within the hypervariable loop of PilE, produces weakly fluorescent cells, indicating that the high density of PilE within the cell envelope is mostly responsible for the strong signal of the cell body. The strong signal of the cell envelope prohibits the detection and analysis of short T4P as explained in the Methods. Moreover, it was unclear how the signal-to-noise ratio affected T4P detection and whether the fluorescent label affects T4P assembly. To assess these potential problems, we counted the average number of T4P per cell. We averaged this number over time (7.5 s) and over multiple cells and found ⟨*N*_*p*_⟩ = 5.9 ± 0.1 (mean ± se) (Figure 1B). We compared this result to the result previously obtained using transmission electron microscopy (TEM) of *N*_*p*_ = 7.2 ± 0.5 (Holz et al., 2010). TEM detects T4P with length ≥ 0.2 μm, indicating that the shortest 20% of T4P are not detected by fluorescence microscopy. However, the difference is small and we conclude that most T4P are detectable by the new method. We note that using both methods we study a projection of T4P projected to 2D.

The number of T4P per cell, *N*_*p*_ = *r*_*p*_τ_*p*_, is determined by the rate at which a cell produces new T4P, r_*p*_, and the lifetime of a T4P, τ_p_. The determination of τ_p_ will be described below. To determine r_*p*,_ newly formed T4P were detected as stepwise increases in fluorescence intensity in the cell contour as described in the Methods. We counted the number of new T4P around the cell contour during a time interval of 7.5 s. We found that the mean rate of T4P production was ⟨*r*_*p*_⟩ = 196 ± 3*min*^−1^ (Figure 1C), i.e., on average a single gonococcus produces 196 new T4P within 1 min. Furthermore, we characterized the distribution of maximum lengths, l_*p*_*^max^*, that T4P reached (Figure 1D). For each T4P, the length was measured at the time point of switching between elongation and retraction. The probability distribution of l_*p*_ agreed well with an exponential function $p\,\left(l_{p}\right)=p_{0}\,e\,x\,p\,\left(-l_{p}/l_{p}^{c}\right)$. A fit to this function yields a characteristic length of $l_{p}^{c}=\left(0.78\pm 0.06\right)$μ*m*. This exponential distribution was expected, assuming that the length of a T4P is determined by the rate of T4P elongation and the detachment rate of the elongation ATPase PilF. If PilF detaches at a constant rate, then its lifetime at the base of the T4P complex is exponentially distributed (Philips et al., 2013) and thus the distribution of T4P lengths is also exponential.

In summary, fluorescence labeling is a useful method for characterizing T4P in *N. gonorrhoeae*. We find that T4P are produced at a high rate of ∼200 T4P min^–1^.

### Elongation and Retraction of Single T4P Proceed Persistently

**FIGURE 2:**
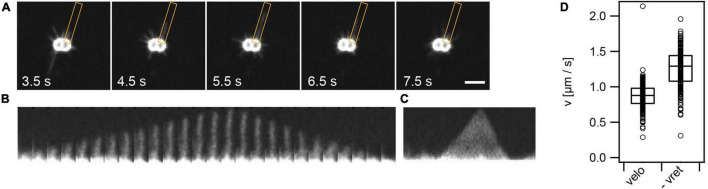
T4P dynamics. **(A)** Time series of T4P dynamics. The orange box denotes the ROI used for analysis of one pilus. **(B)** For analysis, each pilus is aligned. Shown is a T4P that elongates and subsequently retracts. Time interval between two images: 0.1 s. **(C)** Kymograph of T4P shown in panel **(B)** as used for analysis of T4P elongation and retraction speed. **(D)** Velocities of T4P elongation, v_*elo*_, and retraction, v_*ret*_. Shown are circles: single T4P data, box: 25/75 percentiles, and median for 171 T4P.

**FIGURE 3:**
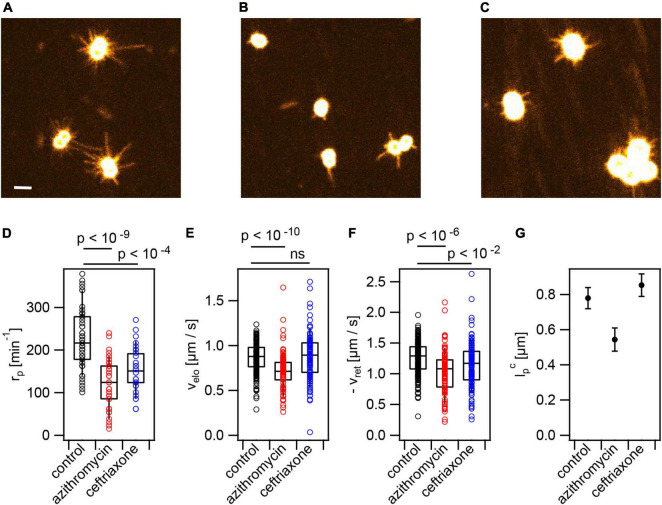
Antibiotic treatment reduces T4P production rate. Cells (Ng226) were treated with azithromycin or ceftriaxone, respectively, at twofold MIC for 3 h. Typical images of fluorescent T4P **(A)** without treatment, **(B)** with azithromycin, **(C)** with ceftriaxone. Scale bar: 2 μm. **(D)** Rate of T4P production, r_*p*_ (>35 cells for each condition). **(E)** T4P elongation velocities, v_*elo*_ (>85 T4P for each condition). **(F)** T4P retraction velocities, v_*ret*_ (>96 T4P for each condition). Shown are circles: single T4P data, box: 25/75 percentiles, and median for 171 T4P. *p*-values obtained from KS test. **(G)** Characteristic maximum T4P lengths $l_{p}^{c}$ and errors obtained from exponential fits to the length distributions.

To further characterize T4P dynamics, we focused on individual T4P (Figure 2 and Supplementary Figure 4). A single T4P was aligned (Figures 2A,B) and a kymograph of this pilus was generated (Figure 2C). We found that T4P elongation and retraction showed little pausing at a time resolution of 50 ms. Furthermore, there were no detectable pauses between T4P elongation and retraction, showing that retraction immediately succeeded elongation. As explained in the Methods part, the kymographs were used for determining the velocities of T4P elongation and retraction (Figure 2D). The elongation velocities were narrowly distributed around *v*_*elo*_ = (0.86 ± 0.02)μ*ms*^−1^ (mean ± standard error) (Figure 3C). The retraction velocities were more broadly distributed around *v*_*ret*_ = (−1.25 ± 0.02)μ*ms*^−1^. We note that the absolute speed measured in this work is lower compared to velocities of $v_{r\,e\,t}^{l\,t}\approx 2\,\mu \,m\,s^{-1}$ determined by laser tweezers (Kurre and Maier, 2012). This difference may be explained by different media used to probe T4P dynamics or by the fact that the fluorescent probe induces friction and thus reduces the retraction speed.

We conclude that in wt gonococci, no pausing is detectable between the end of T4P elongation and the start of T4P retraction. The rate of retraction is higher compared to the rate of elongation in agreement with recent results obtained for *P. aeruginosa* (Koch et al., 2021).

### Treatment With Azithromycin or Ceftriaxone Reduces the Rate of T4P Production

Application of antibiotics may affect T4P production and dynamics and consequentially cellular attraction within colonies. Previously, we have shown that increased attraction enhances survivability under antibiotic treatment (Cronenberg et al., 2021). Here, we will assess the effects of azithromycin and ceftriaxone on T4P production and dynamics. Azithromycin inhibits translation and ceftriaxone inhibits cell wall synthesis.

Cells were incubated at twofold minimal inhibitory concentration (MIC, Supplementary Table 3) for 3 h with the antibiotics prior to imaging. Under these conditions, the pilus production rate was reduced ∼ twofold to $\left\langle r_{p}^{a\,z\,i}\right\rangle =\left(121\pm 8\right)\,m\,i\,n^{-1}$ under azithromycin treatment and to $\left\langle r_{p}^{c\,e\,f}\right\rangle =\left(152\pm 8\right)\,m\,i\,n^{-1}$ under ceftriaxone treatment (Figures 3A–D). This is consistent with early TEM studies showing reduced piliation in response to azithromycin treatment (Gorby and Mcgee, 1990).

At the level of single T4P, azithromycin significantly reduced the speed of T4P elongation to and of retraction to $v_{e\,l\,o}^{a\,z\,i}=\left(0.71\pm 0.02\right)\,\mu \,m\,s^{-1}$ and $v_{r\,e\,t}^{a\,z\,i}=\left(-1.04\pm 0.03\right)\,\mu \,m\,s^{-1}$, respectively (Figures 3C,D and Supplementary Movie 2). Moreover, the characteristic length of T4P was reduced under azithromycin treatment (Figure 3E). Ceftriaxone treatment did not affect the speed of T4P elongation and the length of T4P and slightly reduced the retraction speed (Figures 3C–E and Supplementary Movie 3).

We assessed whether other antibiotics showed similar effects on the T4P dynamics. Ciprofloxacin and streptonigrin are inhibitors of DNA replication. We applied both antibiotics at twofold their MICs. Both antibiotics strongly reduced the rate of T4P production (Supplementary Figure 8). Furthermore, their application reduced the speeds of T4P elongation and retraction slightly but significantly.

We conclude that treatment with antibiotics targeting translation, cell wall synthesis, and DNA replication strongly reduce piliation and interfere with T4P dynamics.

### Treatment With Azithromycin Downregulates Expression of Genes Related to T4P Biogenesis

Since we found the azithromycin and ceftriaxone reduce the rate of T4P production, we addressed the question whether genes involved in T4P biogenesis were downregulated during antibiotic treatment. The transcriptome was sequenced and RNA levels under antibiotic treatment were compared to control samples that were treated with solvent only.

**FIGURE 4:**
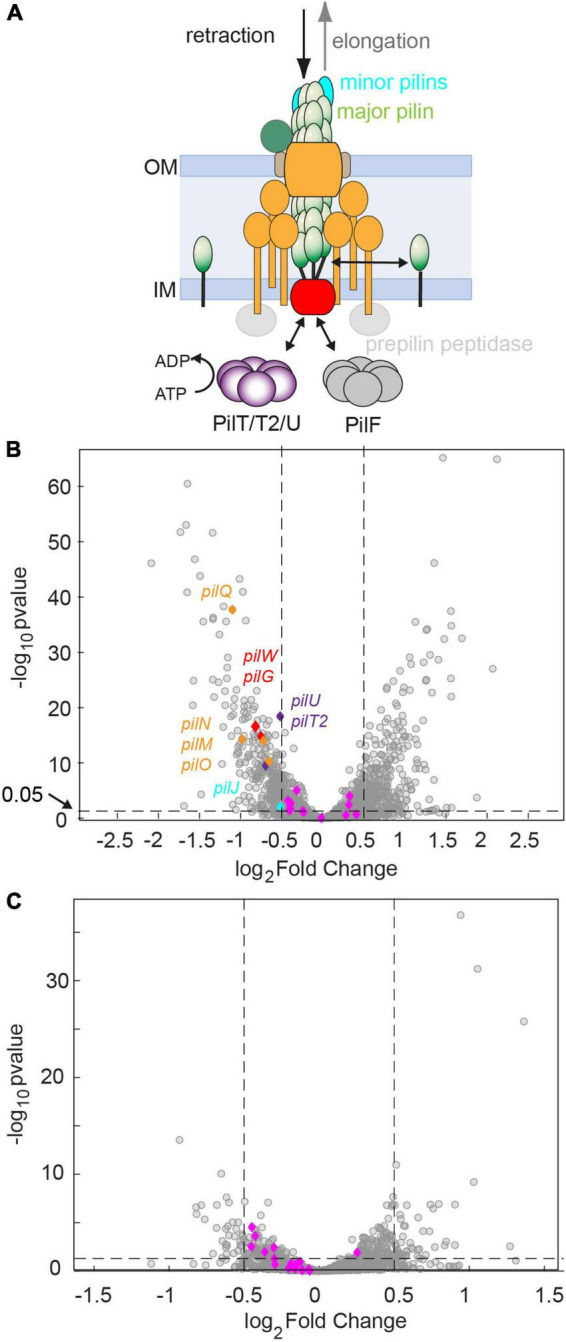
Genes required for assembling the T4P core complex are downregulated after 60 min of antibiotic treatment at twofold MIC. **(A)** Sketch of the T4P assembly complex including the pilus fiber and the elongation and retraction ATPases. **(B,C)** Volcano plots showing adjusted *p*-values as a function of the log_2_fold changes of RNA levels relative to the DMSO-untreated control determined by RNA sequencing. Gray: all genes. Blue and other colors: genes involved in T4P assembly. Dotted lines: Genes are defined to be differentially regulated if adj. *p* ≤ 0.05 and |*log*_2_*foldchange*|≤0.5. **(B)** Treatment with azithromycin. 12.9% of genes are downregulated. 14.3% of genes are upregulated. **(C)** Treatment with ceftriaxone. 1.6% of genes are downregulated. 2.6% of genes are upregulated.

At the genome-wide level, azithromycin treatment caused differential regulation of a large fraction of genes. After 60 min of treatment, 12.9% were downregulated (at least −0.5 log_2_ fold or 0.7 fold change in mRNA levels, adjusted *p*-value of ≤ 0.05) and 14.3% were upregulated (at least 0.5 log_2_ fold or 1.4 fold change in mRNA levels, adjusted *p*-value of ≤ 0.05) (Figure 4B). Fewer genes were affected after 60 min of ceftriaxone treatment, with 1.6% downregulation and 2.6% upregulation (Figure 4C).

We investigated how azithromycin and ceftriaxone treatment affect mRNA levels of the 22 genes encoding for proteins that assemble the T4P core complex and the T4P fiber (Figure 4A). The genes considered as T4P assembly genes, listed in Supplementary Data Sheet 2, were annotated manually. After 60 min of azithromycin treatment, we find that 9 out of 22 genes were downregulated (Supplementary Data Sheet 2 and Figure 4B) resulting in an enrichment of this group of genes (*p* = 0.001, one-sided Fisher’s exact test). None of the T4P assembly genes were upregulated. Most prominently, the *pilMNOPQ* operon (with the exception of *pilP*) was downregulated (Figures 4A,B). This operon encodes for PilQ proteins forming the secretin for the extrusion of the pilus fiber and PilMNOP proteins assembling the periplasmic complex. Moreover, the gene encoding the platform protein, PilG, residing within the cytoplasmic membrane, is downregulated. Next to genes involved in core complex assembly, the expression of a gene affecting piliation, *pilW*, and the minor pilin *pilJ* is reduced. Finally, the retraction ATPases PilT2, and PilU are downregulated. Under ceftriaxone treatment, none of the T4P-related genes is differentially regulated under our criteria (adj. *p*-value ≤ 0.05 and | log2-fold change| ≥ 0.5). Yet multiple genes meet the adj. *p*-value ≤ 0.05 criterium, suggesting that they are weakly downregulated (Figure 4C and Supplementary Data Sheet 2).

To summarize, treatment with azithromycin downregulates T4P biogenesis genes in agreement with lower rates of T4P production observed by fluorescence microscopy.

### Other External Stresses Reduce the Rate of T4P Production and T4P Dynamics

**FIGURE 5:**
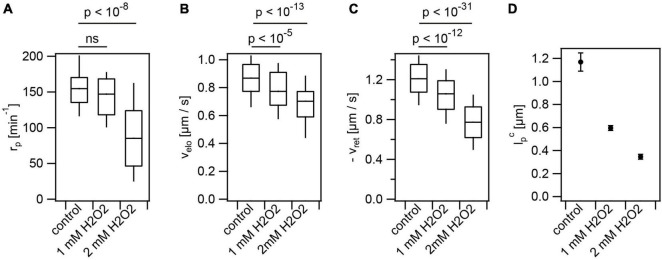
Hydrogen peroxide reduces T4P production rate and T4P dynamics. Cells (Ng226) were treated with H_2_O_2_ for 15 min. **(A)** Rate of T4P production, r_*p*_. (>40 cells for each condition) **(B)** T4P elongation velocities, v_*elo*_ (>97 T4P for each condition). **(C)** T4P retraction velocities, v_*ret*_ (>100 T4P for each condition). Shown are box: 25/75 percentiles, and median for 171 T4P. *p*-values obtained from KS test. **(D)** Characteristic maximal T4P length. (>340 T4P for each condition).

During their infection cycle, *N. gonorrhoeae* experiences various stresses. For example, lactobacilli produce H_2_O_2_ and lactic acid (Gong et al., 2016); macrophages produce reactive oxygen species including H_2_O_2_ (Quillin and Seifert, 2018). We assessed the effects of these stresses on T4P production and dynamics. Cells were pre-incubated with hydrogen peroxide for 15 min prior to imaging. Please note that the incubation time was shorter compared to the incubation time with antibiotics of 3 h. Therefore, the control values are slightly different from Figures 1, 3. With 2 mM H_2_O_2_, the rate of T4P production was reduced to (91 ± 8)*min*^−1^ (Figure 5A and Supplementary Movie 4). The speeds of T4P elongation and retraction were reduced to $v_{e\,l\,o}^{H\,2\,O\,2}=\left(0.68\pm 0.02\right)\,\mu \,m\,s^{-1}$ and $v_{r\,e\,t}^{H\,2\,O\,2}=\left(-0.78\pm 0.02\right)\,\mu \,m\,s^{-1}$ (Figures 5B,C), respectively. H_2_O_2_ showed the strongest effect on the characteristic maximal length of T4P which was reduced from $l_{p}^{c}=\left(1.15\pm 0.05\right)\,\mu \,m$ to $l_{p}^{c,H\,2\,O\,2}=\left(0.35\pm 0.02\right)\,\mu \,m$ (Figure 5D).

**FIGURE 6:**
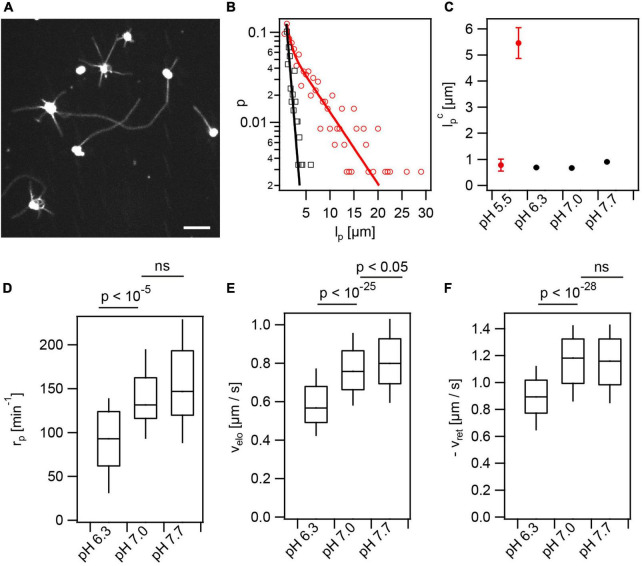
Extracellular pH affects T4P dynamics. In the presence of lactic acid, the external pH was adjusted to different values. **(A)** At pH 5.5 T4P appear elongated. Scale bar: 5 μm. **(B)** Distribution of maximal T4P lengths at pH 5.5 with double exponential fit (red) and pH 7.0 with exponential fit (black). **(C)** Characteristic maximal T4P length at varying pH. The characteristic length was determined from an exponential fit for pH 6.3–7.7. At pH 5.5, the two characteristic lengths obtained from the double-exponential fit are shown. (>247 T4P for each condition) **(D)** Rate of T4P production, r_*p*_ (>32 cells for each condition). **(E)** T4P elongation velocities, v_*elo*_ (>87 T4P for each condition). **(F)** T4P retraction velocities, v_*ret*_ (>100 T4P for each condition).

Furthermore, we addressed the effects of lactic acid and external pH on T4P dynamics. During their infection cycle, gonococci experience pH ranging from pH 4–8 (Pettit et al., 1999). We supplemented the media with 100 mM lactic acid, since this acid is produced by lactobacilli known to counteract gonococcal infections (O’Hanlon et al., 2013). Prior to T4P fluorescence labeling, the pH of the solution was adjusted to different pH by titration of sodium hydroxide (NaOH). The same pH was maintained during imaging. So far, experiments were performed at pH 7.0. We found that increasing the pH to pH 7.7 showed little effect on T4P dynamics (Figures 6D–F). By contrast, decreasing the pH to pH 6.3 reduced the rate of T4P production, and the speeds of T4P elongation and retraction (Figures 6D–F and Supplementary Movie 5). At pH 5.5, we observed that the lengths of some T4P reached beyond 30 μm (Figures 6A,B). At this pH, T4P did not retract. The distribution of T4P lengths could not be described by a single exponential function like the distributions at higher pH. Instead, we fitted a double exponential function (Figure 6B) with one characteristic length of $l_{p\,1}^{c,p\,H\,5.5}=\left(0.8\pm 0.2\right)\,\mu \,m$ that was comparable to the length at higher pH and a second characteristic length of $l_{p\,2}^{c,p\,H\,5.5}=\left(5.5\pm 0.6\right)\,\mu \,m$ (Figure 6C). This finding indicates that PilT is inactive or its binding to the T4P complex is inhibited at pH 5.5 while PilF is still functional. We suggest that consecutive PilF binding events generate the long T4P while the short T4P result from single binding events and, therefore, the lengths of the latter T4P are comparable to the length at higher pH.

Finally, we investigated how energy depletion affects T4P dynamics, in particular the effect of the proton motive force. We applied the uncoupler CCCP for 10 min and found that the T4P production rate as well as single T4P dynamics were strongly reduced (Supplementary Figure 9). This finding is consistent with earlier studies of T4P retraction, where we showed that depletion of pmf reduces the speed of T4P retraction (Kurre et al., 2013). Interestingly, the strong and rapid reduction of the T4P production rate can explain why gonococcal colonies disassemble under CCCP treatment (Dewenter et al., 2015).

Taken together, we found that various external stresses including hydrogen peroxide, uncouplers of the membrane potential, and low pH reduce the rate of T4P production and reduce the rates of T4P elongation and retraction. The only exception was application of low pH, where we found that T4P retraction was inhibited leading to elongated T4P.

### The Rate of T4P Production Is Independent of T4P Lifetime but Correlates With T4P Density

We found that application of a variety of different stresses reduces the rate of T4P production r_*p*_. This reduction may be caused either by increased lifetime of T4P τ_p_, which would reduce the turn-over of T4P, but may not affect the number of T4P, N_*p*_. On the other hand, if the production rate doesn’t depend on the lifetime, then we expect that lower production rate results in a lower N_*p*_.

**FIGURE 7:**
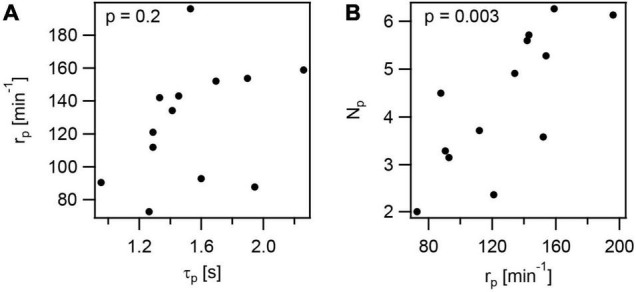
Correlations in T4P dynamics and production rate. The data plotted are mean values from independent experiments at the different conditions investigated in this study. **(A)** Rate of T4P production as a function of the T4P lifetime. **(B)** Rate of T4P production as a function of the number of T4P per cell. *p*-values were obtained from 2-tailed test for the hypothesis that there is no correlation.

To assess whether r_*p*_ correlates with τ_p_, we determined the T4P lifetimes as τ_*p*_ = *l*_*p*_/*v*_*elo*_ + *l*_*p*_/*v*_*ret*_. Depending on the stress conditions, the lifetime varied between τ_*p*_ = (1.0 ± 0.1)*s* and τ_*p*_ = (2.3 ± 0.1)*s* (Supplementary Figure 10). The T4P production rate r_*p*_ and T4P lifetime τ_p_ showed no significant correlation (Figure 7A and Supplementary Table 4). Next, we determined the mean number of T4P per cell, N_*p*_. This number is reduced under all stress conditions. In particular, N_*p*_ was reduced ∼ threefold under azithromycin and CCCP treatment (Supplementary Figure 10B). We found strongly significant correlation between N_*p*_ and r_*p*_ (Figure 7B and Supplementary Table 4), indicating that the reduced rate of T4P production reduces the density of T4P.

It is also interesting to note that the velocities of T4P elongation and retraction show very strong correlation (*r* = 0.9 ± 0.2,Supplementary Table 4). This indicates that reduction of the rates at which PilF and PilT work are similar under different stress conditions, possibly due to reduced energy levels.

We conclude that the lifetime of T4P is variable under different stress conditions and does not correlate with the rate of T4P production. Reduction of the T4P production rate results in reduced number of T4P in agreement with reduced expression of genes involved in the T4P complex under antibiotic treatment.

## Discussion

### Putative Functions of Fast T4P Dynamics in *N. gonorrhoeae*

Dynamics of T4P have been characterized for different types of pilus systems in various species (under laboratory conditions) revealing a large dynamic range. For example, *P. aeruginosa* produces no more than ∼8 T4P min^–1^ (Koch et al., 2021) and *V. cholerae* competence pili are produced at ∼1 T4P min^–1^ (Ellison et al., 2018). Here we show that the rate of T4P production of ∼200 T4P min^–1^ in *N. gonorrhoeae* is very high compared to these bacterial species. This rate is likely underestimated, because fluorescently labeled pilin in the membrane produce a strong signal and prohibit the characterization of short T4P.

Moreover, gonococcal T4P generate high force by retraction on the order of ∼150 pN (Marathe et al., 2014) compared to ∼40 pN of *P. aeruginosa* (Ribbe et al., 2017) and ∼10 pN of *V. cholerae* (Ellison et al., 2018). We propose that high forces and fast T4P dynamics are required for the tug-of-war that enables twitching motility both at surfaces (Marathe et al., 2014) and within colonies (Bonazzi et al., 2018; Welker et al., 2018; Zollner et al., 2019) formed by *N. gonorrhoeae* and closely related *N. meningitidis*. In particular, within colonies, high force and fast dynamics enable fluid-like behavior of colonies. The fluid-like state is important for colonization of blood vessels (Bonazzi et al., 2018) and survivability within colonies (Cronenberg et al., 2021). Motor activity also enables gonococcal colonies to respond rapidly to environmental changes like oxygen limitation (Dewenter et al., 2015); when oxygen levels fluctuate close to a threshold concentration, T4P dynamics drive colony disassembly and re-assembly within seconds.

Taken together, we propose that fast T4P dynamics and high force generation support the lifestyle of *Neisseria*. A high density of T4P enables rapid colony formation, but the T4P-bonds are highly dynamic and thus keep colonies in a fluid-like state.

### Downregulation of T4P-Related Genes Agrees With Reduction of T4P Production Rate Under Azithromycin Treatment

To better understand the effect of antibiotic treatment on T4P production, we characterized the transcriptional response of T4P-related genes to antibiotic treatment, focussing on two drugs that are currently used for treatment of gonorrhea, namely azithromycin and ceftriaxone. We found that genes required for building the membrane-standing T4P complex, are strongly downregulated under azithromycin treatment. The effect of downregulation was most severe (twofold) for *pilQ* and other members of the *pilMNOPQ* operon (Pelicic, 2008). Similarly, the expression of the gene encoding the platform protein PilG and a gene affecting piliation (*pilW*) (Carbonnelle et al., 2005; Hu et al., 2020) are downregulated. Thus, the reduced expression of genes encoding for the membrane-standing T4P complex reduces the number of complexes from which T4P can extrude, explaining why the T4P density is reduced. Regulation of T4P related genes in *N. gonorrhoeae* is poorly understood. In the following, we will consider the potential roles of known regulators whose expression is significantly changed in our study. Most interestingly, *misR*, a homologue of *E. coli cpxR*, is part of a two-component system involved in response to cell envelope stress (Kandler et al., 2016). Its deletion causes downregulation of genes involved in T4P production (|log_2_fold change| ≥ 0.5, *p* ≤ 0.05), including *pilQ*, *pilP*, *pilO*, *pilW*, *pilG*, *pilD*, *pilU*, *pilIJKL* (McClure et al., 2020). The differential expression similar to our results for azithromycin treatment. Therefore, we assessed the expression of *misR* (NGFG_00314) and found 0.7 fold downregulation. In another study, MtrR was shown to be a repressor of *pilM* (Folster et al., 2007). For this regulator, we find no significant change in its transcription under antibiotic treatment.

In summary, downregulation of T4P-related genes during antibiotic treatment explains why the rate of T4P production is reduced.

### Putative Roles of Metabolic Changes in T4P Dynamics

The T4P elongation and retraction ATPases rely on ATP, and therefore, reduction of ATP levels will influence their activities. We have shown previously that depletion of ATP reduces the speed of gonococcal twitching motility (Kurre et al., 2013). The speed of twitching motility tightly correlates with the speed of T4P retraction (Marathe et al., 2014), indicating that reduced ATP levels reduce the speed of T4P retraction. Furthermore, the velocity of T4P retraction depends on proton motive force (Kurre et al., 2013). Here, we found that azithromycin and ceftriaxone treatment reduced the velocity of T4P retraction and azithromycin additionally reduces the velocity of T4P elongation.

**FIGURE 8:**
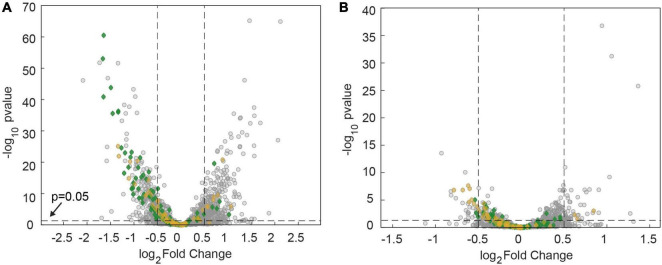
Downregulation of metabolic genes after 60 min of antibiotic treatment at twofold MIC. Volcano plots showing adj. *p*-values as a function of the log_2_fold changes of RNA levels relative to the DMSO-untreated control determined by RNA sequencing. Gray: all genes. Green: genes involved in carbohydrate metabolism. Orange: Genes involved in energy metabolism. **(A)** Treatment with azithromycin. **(B)** Treatment with ceftriaxone.

We addressed the question whether antibiotic treatment affected metabolic pathways that govern the energy levels in gonococci. Analyzing the transcriptomes based on 17 categories from KEGG orthology (McClure et al., 2020), we found that carbohydrate metabolism was differentially regulated under azithromycin treatment (Figure 8A) (*p* = 9×10^−6^, one-sided Fisher’s exact test with Bonferroni multiple testing correction). Most of these genes are downregulated (Supplementary Data Sheet 3). In particular, genes involved in ATP-producing glycolysis were expressed at lower levels. Under ceftriaxone treatment, energy metabolism was significantly affected (Figure 8B) (*p* = 0.001, one-sided Fisher’s exact test with Bonferroni multiple testing correction). Again, most genes in this category were downregulated. This category involves oxidative phosphorylation which generates both ATP and proton motive force. Taken together, transcriptomic analysis suggests that the energy levels are reduced by treatment with azithromycin and ceftriaxone.

In this study, we also investigated whether depletion of proton motive force (using the uncoupler CCCP) phenotyped the application of stressors. We found that reduction of piliation can be achieved by depleting the proton motive force. Furthermore, reduced proton motive force and ATP levels would reduce the velocity of T4P retraction (Kurre and Maier, 2012; Kurre et al., 2013) in agreement with our results.

In summary, azithromycin and ceftriaxone treatments cause downregulation of different sets of metabolic genes that signify reduced energy levels. Depletion of proton motive force phenotypes the application of stressors. This strongly suggests that energy depletion caused by application of stressors is involved in reduction of T4P dynamics.

### Gonococcal Piliation Is Reduced Under a Large Variety of Stresses Including Antibiotics With Different Targets, Hydrogen Peroxide, and Lactic Acid

Previously, it was shown that production of T4P enhances tolerance against hydrogen peroxide (Stohl et al., 2013) and ceftriaxone (Wang et al., 2018). Moreover, we showed that increasing the T4P-mediated attractive forces conferred higher survivability under ceftriaxone treatment (Cronenberg et al., 2021). Therefore, we expected that upregulation of pilus production can protect gonococci from antibiotic stress. To the contrary, we found that under all stress conditions tested in this study, the T4P production rate and T4P density were reduced. For *N. meningitidis*, further treatments have been shown to reduce piliation, providing additional examples for stress-induced reduction in T4P levels. Phenothiazines reduce piliation by interfering with the sodium motive force (Denis et al., 2019) and other therapeutic compounds reduce T4P formation by inhibiting the elongation ATPase (Aubey et al., 2019). It is tempting to speculate that the optimal rate of T4P production in the presence of external stress follows a trade-off between the protective effect of strong cell-to-cell attraction at high T4P density (Cronenberg et al., 2021) and the reduction of drug uptake rates by *pilQ* downregulation (Nandi et al., 2015). Interestingly, PilQ was reported to be involved in uptake of antibiotics (Chen et al., 2004; Nandi et al., 2015). Specific mutations in PilQ either enhanced (Chen et al., 2004) or decreased (Nandi et al., 2015) antibiotic susceptibility. Deletion of *pilQ* decreased antibiotic susceptibility, suggesting that PilQ plays a role in antibiotic permeation (Nandi et al., 2015). Downregulation of *pilQ* expression would therefore increase antibiotic tolerance and could be a gonococcal response to antibiotic treatment. Since *pilQ* is part of the *pilMNOPQ* operon it is conceivable that the entire membrane-spanning complex built by the corresponding proteins are required for antibiotic permeation as well, although there is currently no evidence for their involvement (Nandi et al., 2015). On the other hand, it is unclear whether reduction of piliation results from a concerted stress response.

In general, reduction of T4P density reduces cell-to-cell attraction. The reduced piliation under azithromycin and ciprofloxacin treatment observed here is in excellent agreement with our previous finding that gonococcal colonies treated with these drugs become more fluid (Cronenberg et al., 2021). However, in that study we also showed that ceftriaxone enhances the attractive interaction and reduces the fluidity of colonies. Here, we observed that ceftriaxone treatment reduces piliation and downregulates genes involved in forming the T4P complex. Quantitatively, these effects are weaker compared to azithromycin treatment, yet their tendency is not in agreement with enhanced attractive force. We conclude therefore, that piliation is not the only factor determining colony fluidity. Instead, changes in surface-exposed structures or local order within the colony [as observed for ceftriaxone treatment (Cronenberg et al., 2021)] might counteract the effect of reduced piliation.

To summarize, piliation of *Neisseria* is reduced under a large variety of stresses, and it will be interesting to test whether a concerted stress response regulates this reduction.

## Conclusion

T4P have been termed the bacterial “swiss army knife” because they are involved in many different functions (Berry and Pelicic, 2015). Here, we investigated the piliation response to a variety of different stresses that gonococci are likely to face. We reveal that under all conditions tested, the rate of T4P production and the number density of T4P are reduced. This suggests that external stress interferes with T4P functionality. In future studies it will be interested to find out whether downregulation of T4P is a general stress response.

## Data Availability Statement

The authors acknowledge that the data presented in this study must be deposited and made publicly available in an acceptable repository, prior to publication. Frontiers cannot accept a manuscript that does not adhere to our open data policies. The data presented in the study are deposited in the NCBI GEO repository, accession number GSE195465.

## Author Contributions

SK-R, IW, IR, JG, and BM designed research. SK-R and IW performed research. SK-R, IW, IR, and JG analyzed data. SK-R, IW, IR, and BM wrote the manuscript. All authors contributed to the article and approved the submitted version.

## Conflict of Interest

The authors declare that the research was conducted in the absence of any commercial or financial relationships that could be construed as a potential conflict of interest.

## Publisher’s Note

All claims expressed in this article are solely those of the authors and do not necessarily represent those of their affiliated organizations, or those of the publisher, the editors and the reviewers. Any product that may be evaluated in this article, or claim that may be made by its manufacturer, is not guaranteed or endorsed by the publisher.
