# Supplementary material for: External Stresses Affect Gonococcal Type 4 Pilus Dynamics
Source: Front Microbiol. 2022 Feb 25;13:839711. doi: 10.3389/fmicb.2022.839711 (PMC8914258; doi:10.3389/fmicb.2022.839711)
Supplement: Supplementary file 1 [file Data_Sheet_1.pdf]

Supplementary information for

External stresses affect gonococcal type 4 pilus dynamics

Sebastian Kraus-Römer[1]*, Isabelle Wielert[1]*, Isabel Rathmann[1], Jan Grossbach[2], Berenike Maier[1, 3, #]

*equal contribution

[1] University of Cologne, Institute for Biological Physics

[2] University of Cologne, Faculty of Mathematics and Natural Sciences, CECAD

[3] Center for Molecular Medicine Cologne

berenike.maier@uni-koeln.de

Supplementary Figures

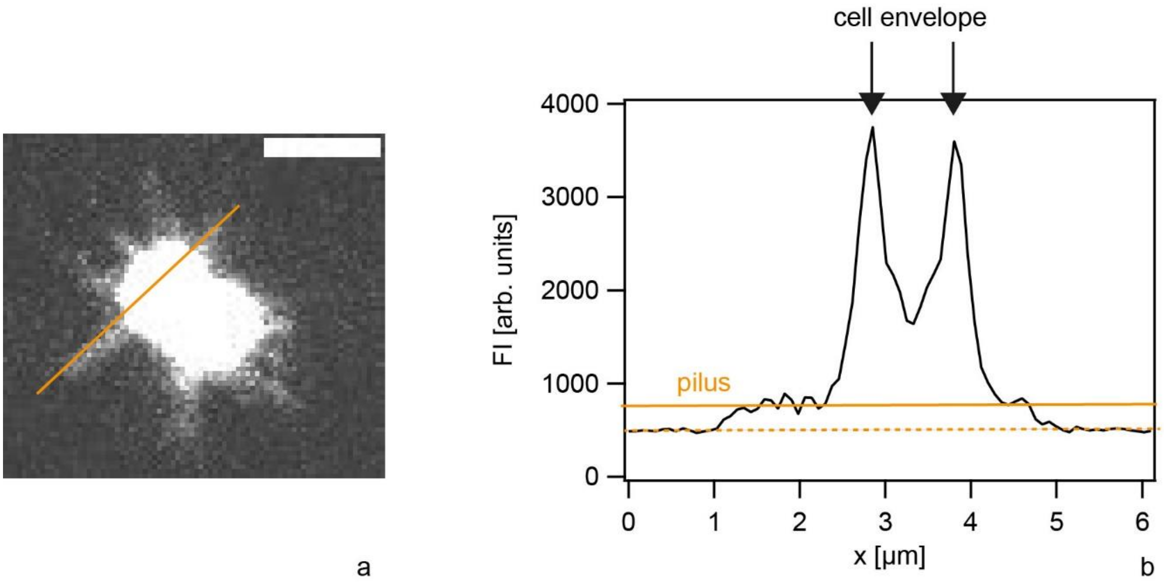

Fig. S1. The fluorescence intensity of the cell contour exceeds the intensity of labeled T4P. a) Labeled gonococcus. The intensity profile is measured along the orange line. Scale bar: 2 µm. b) The intensity profile along the orange line of a). The background intensity is identified (dashed orange line at 500 a.u). The labelled T4P intensity was 750 a.u. (solid orange line). The cell body was found to have an intensity maximum of 3800 a.u.

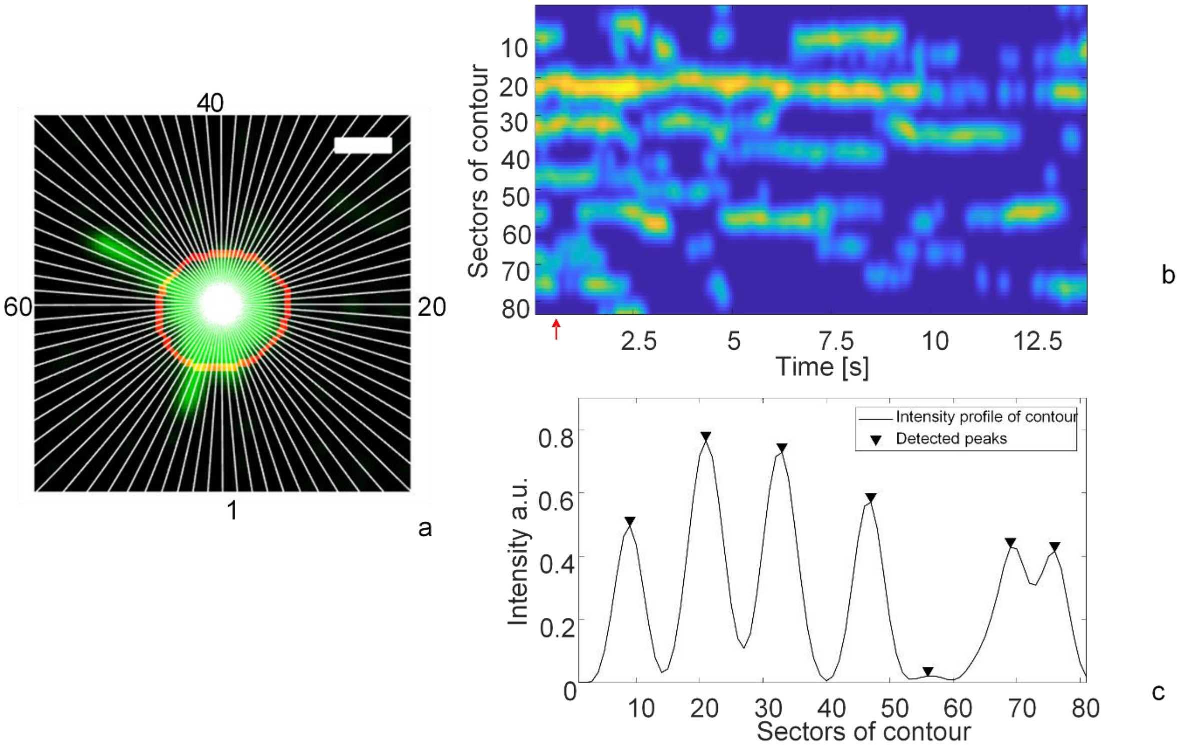

Fig. S2 Determination of the T4P production rate, r_P. a) Labeled gonococcus with T4P (green) and its contour (red). The white lines represent the division of the contour in 80 parts. Scale bar: 1 µm. b) Kymograph of contour. Frame 10 marked with red arrow. c) Intensity profile from contour for frame 10 of the kymograph with detected peaks (black arrows) representing the present T4P.

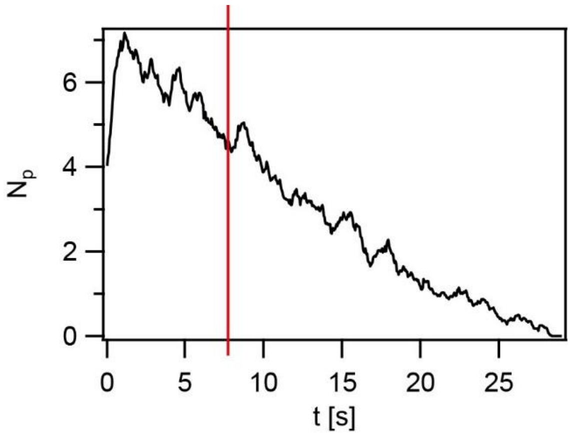

Fig. S3 The detected number of T4P per cell, N_p, decreases rapidly. N_p as a function of time averaged for 40 gonococci. At time t = 0, image acquisition starts. The red line marks the initial 7.5 s.

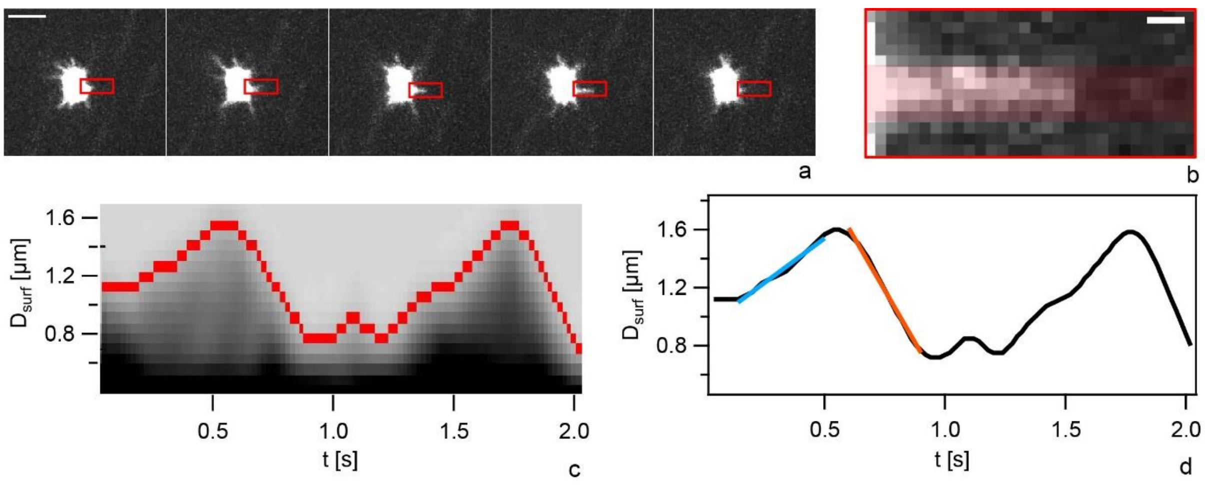

Fig. S4 Determination of the velocities of T4P elongation, v_{elo}, and retraction, v_{ret}. a) Time lapse of T4P elongation and retraction. Time between each image: 0.5 s, Scale bar: 3 µm. b) ROI shown in a). The red shaded area represents area used to create the intensity profiles for the kymograph. Scale bar: 0.3 µm. c) Kymograph and edge detection. Distance of the cell surface, D_{surf}, as a function of time. d) Example track of the pilus tips' distance to the cell surface D_{surf} with time. Blue line: slope used to determine the elongation velocities. Orange line: slope used to to determine the retraction velocities.

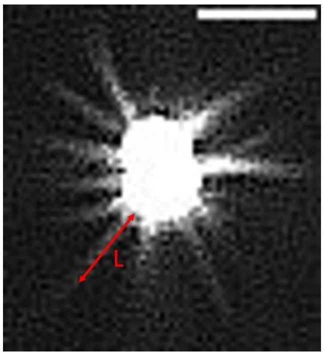

Fig. S5 Determination of the maximum T4P length, l_p. The sum of all intensity values for the first 100 frames of the acquired videos. The red line represents how the length L of the T4P are determined. Scale bar: 2 µm.

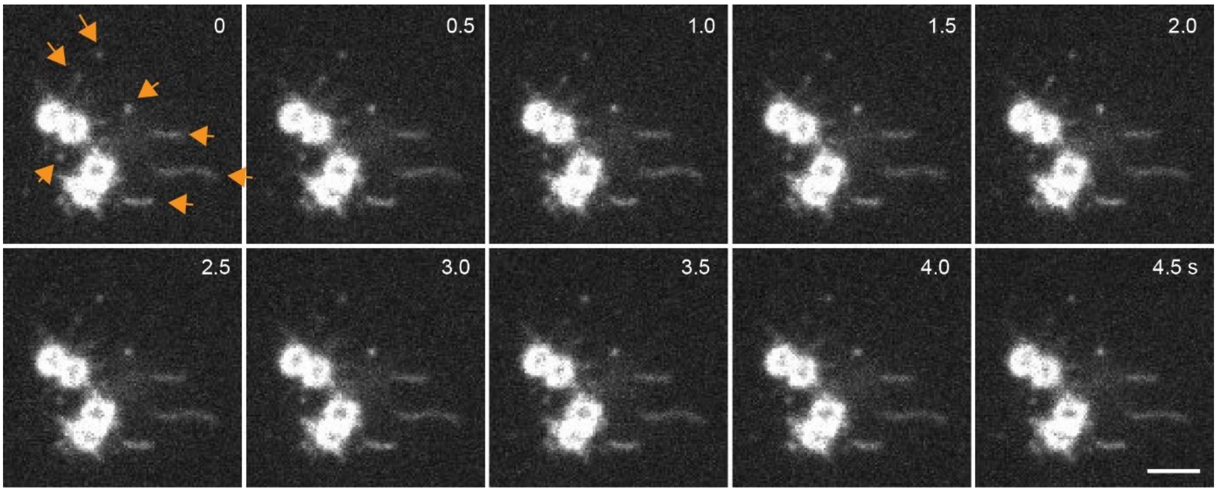

Fig. S6 Time lapse of *ΔpilT* mutant gonococci (Ng250). Orange arrows depict typical fluorescent foci observed around *ΔpilT* bacteria, indicating that mostly the tips of T4P are fluorescent. We suggest that fluorescent pilin is integrated into the T4P during and shortly after incubation with the fluorescent dye. After removal of AF488 molecules, new (unlabeled) pilin is synthesized and integrated into existing T4P.

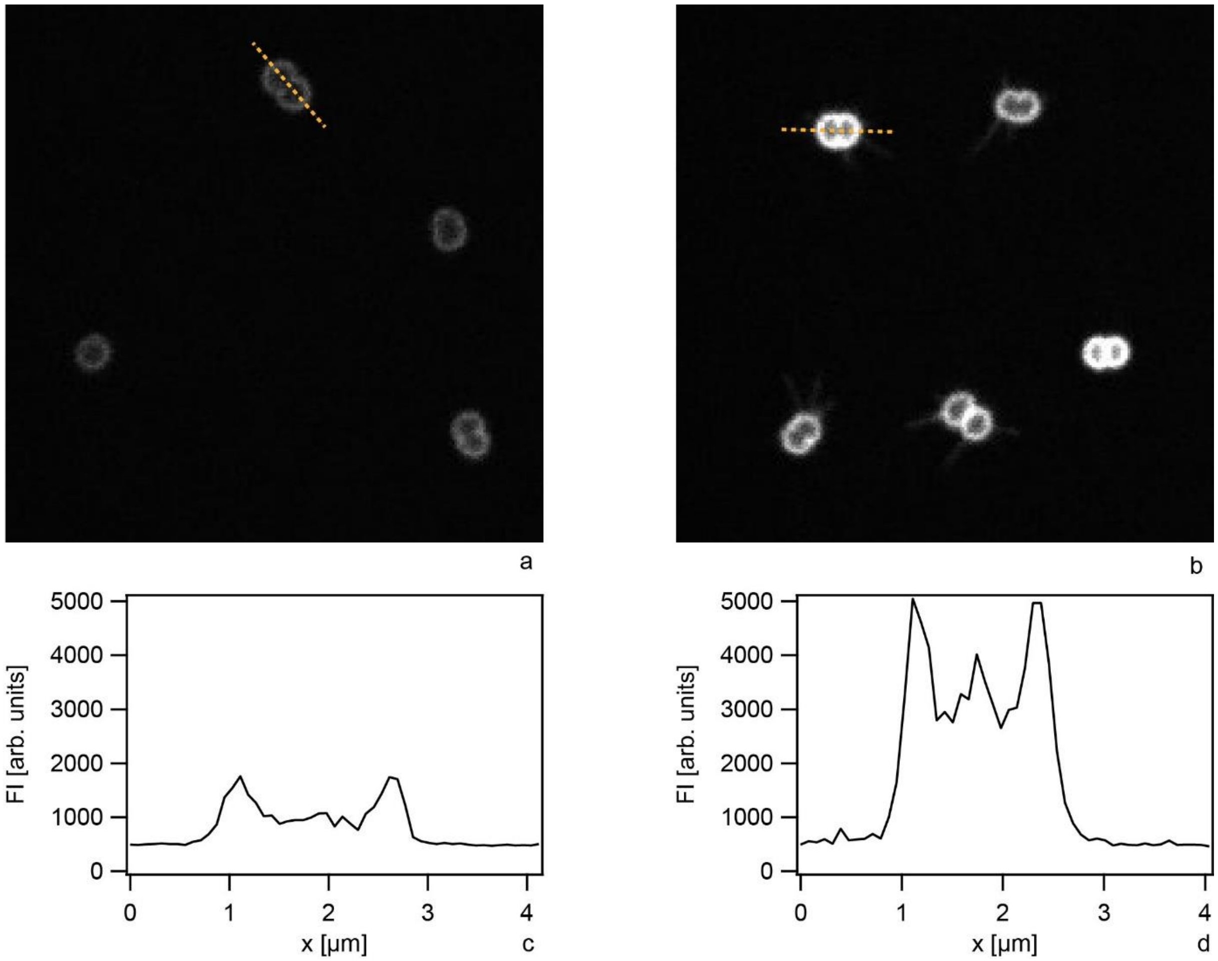

Fig. S7 Staining of the cell envelope. Bacteria were incubated with Flour 488 C_5 maleimide dye as described in the Methods. a) Parental strain NG150 lacking the T126C modification and b) NG226 carrying the T126C modification in the major pilin. c) Intensity profile through the dotted lines shown in a). d) Intensity profile through the dotted lines shown in b).

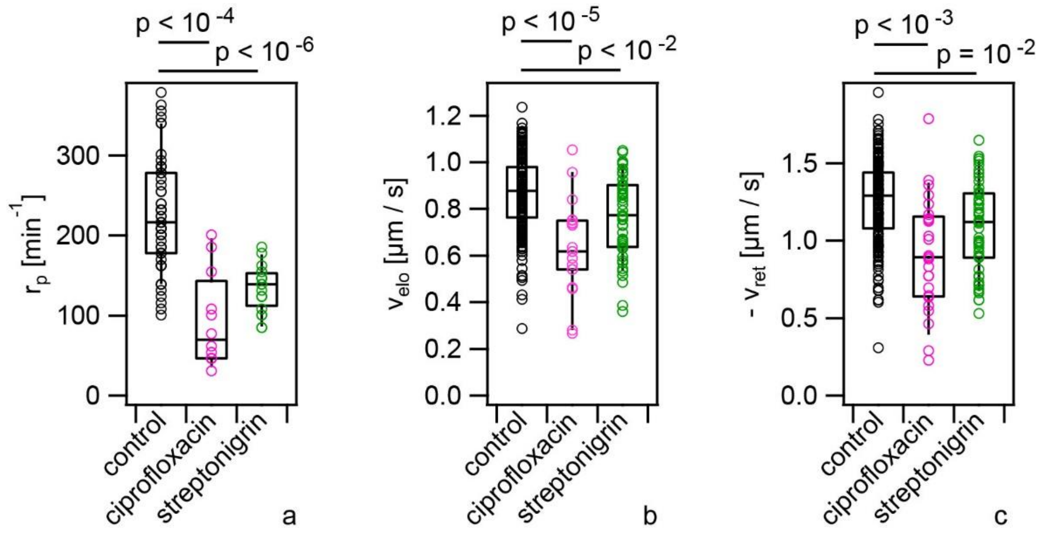

Fig. S8 Antibiotics that interfere with DNA replication reduce T4P production rate. Cells were treated with ciprofloxacin or streptonigrin, respectively at 2-fold MIC for 3 h. a) Rate of T4P production, r_p. (> 12 cells for each condition) b) T4P elongation velocities, v_{elo} (> 18 T4P for each condition). c) T4P retraction velocities, v_{ret} (> 24 T4P for each condition). Shown are circles: single T4P data, box: 25 / 75 percentiles, and median for 171 T4P. p-values obtained from KS test.

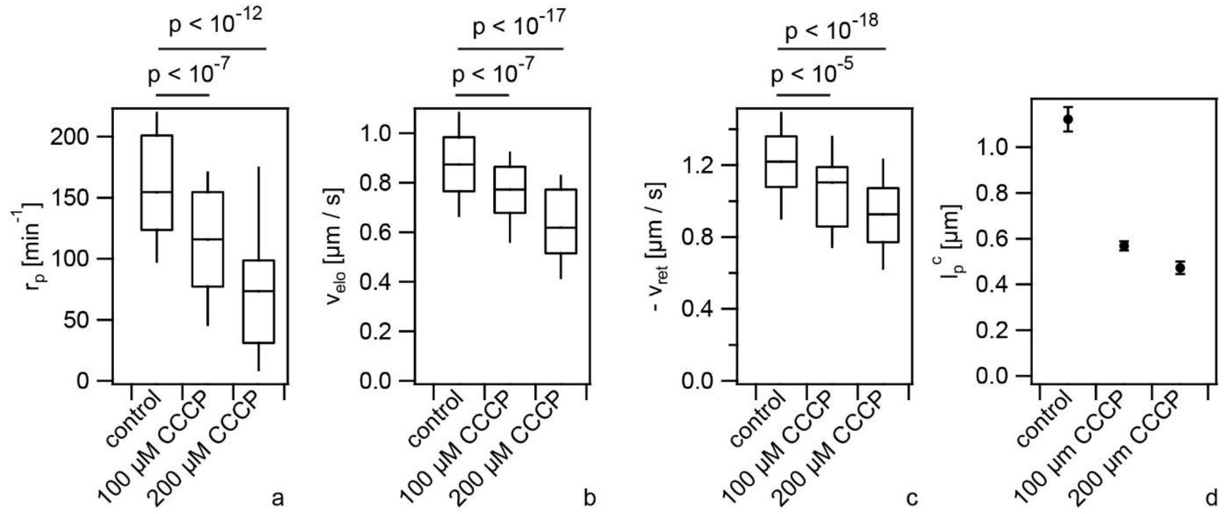

Fig. S9 CCCP reduces T4P production rate and T4P dynamics. Cells were treated with CCCP for 10 min. a) Rate of T4P production, r_p. (> 30 cells for each condition) c) T4P elongation velocities, v_{elo} (> 87 T4P for each condition). d) T4P retraction velocities, v_{ret} (> 100 T4P for each condition). Shown are box: 25 / 75 percentiles, and median for 171 T4P. p-values obtained from KS test. d) Characteristic maximal T4P length. (> 156 T4P for each condition)

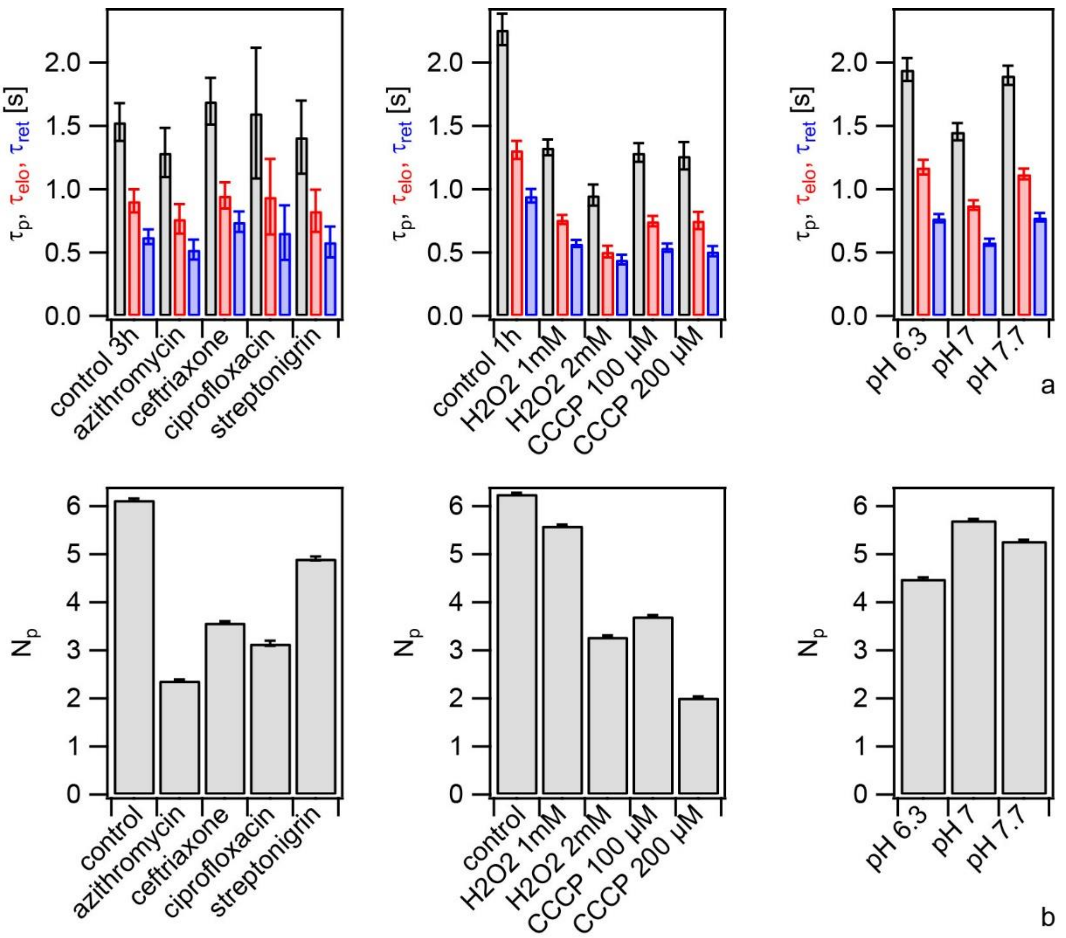

Fig. S10 T4P lifetimes, τ_p, and number of T4P, N_p, under the different conditions studied. a) Mean T4P lifetimes calculated as $\tau_p = l_p/v_{elo} + l_p/v_{ret}$ (grey), mean durations of T4P elongation calculated as $\tau_{elo} = l_p/v_{elo}$ (red), and mean durations of T4P elongation calculated as $\tau_{ret} = l_p/v_{ret}$ (blue). Error bars: standard error of the mean. b) Mean number of T4P per cell, N_p, and standard error. Please note that the incubation times in the different subpanels are different (see Methods).

Supplementary Tables

***Neisseria gonorrhoeae* strains**	**relevant genotype**	**source**
ΔG4 (Ng150)	*G4::aac*	(1)
pilE^{T126C}(Ng226)	*G4::aac*<br>*pilE::pilE*^{T126C}	this study
pilE^{T126C} Δ*pilT* (Ng250)	*G4::aac*<br>*pilE:: pilE*^{T126C}<br>*pilT::m-Tn3cm*	this study, (2)

Table S1 Strains used in this study

name	5'→3' sequence
sk5	CCGCTCGAGCGGTTCCGACCCAATCAACACACC
sk9	TATAACCCTCTTTATTTTTTCCTCC
sk10	AGCTTTGGCTAACACACACG
sk32	GGGCCTTGAAGCGCAATCGATATA
sk40	AATATCTATACTTAAGTCATTTGGCATCAGATGCCTTA
sk43	AAAATGGTTCTGCGGACAGCCGGTT**TGC**CGCGCCGCCAAAGACGACGACGCCG
sk44	ACCGAACCGTTTTCACGCTTGGCCCACAGGGAGAG
sk45	CAACCCTTAAAGGAAAAACCATGCAATAC
sk129	TTCCGACCCAATCAACACA
sk131	TTTAAGGCCTAATTTGCCTCATTTGGCATCAGATGCCTTAT
sk132	ATCTGATGCCAAATGAGGCAAATTAGGCCTTAAATTTTA
sk135	GATTTATTTAAAATTTAAGGCCTAATTTGCCAATTCCCGACTGATTGTGAGG
sk143	GTCGGGAATTGGCAAATTAGGCCTTAAATTTTAAATAAATC
sk145	ATGCCGTCTGAATAGTCGAATCGATGCTGTG
sk146	ATCTGATGCCAAATGACCCGGTGCTTCATCACC
sk147	CACCGGGTCATTTGGCATCAGATGCCTTA
sk158	GTATCGGCAATGACGGTTCG

Table S2 Primers used in this study

Substance	MIC		Source
	Δ*G4* *(Ng150)*	ΔG4 *pilE*^{T126C} (Ng226)	
azithromycin[µg/mL]	0.128	not determined	(3)
ceftriaxone[µg/mL]	0.008	not determined	(3)
cirpofloxacin[µg/mL]	0.004	0.004	this study
streptonigrin[µg/mL]	0.064	0.064	this study
H_2O_2 [mM]	2	1	this study

Table S3 Minimal inhibitory concentrations

	l_p	N_p	v_{elo}	v_{ret}	τ_p
r_p	$r = 0.7 \pm 0.2$ $p = 0.01$	$r = 0.8 \pm 0.2$ $p = 0.003$	$r = 0.9 \pm 0.1$ $p = 3 \cdot 10^{-5}$	$r = 0.9 \pm 0.1$ $p = 10^{-5}$	$r = 0.4 \pm 0.3$ $p = 0.2$
l_p		$r = 0.6 \pm 0.2$ $p = 0.02$	$r = 0.7 \pm 0.2$ $p = 0.01$	$r = 0.8 \pm 0.2$ $p = 0.003$	
N_p			$r = 0.6 \pm 0.2$ $p = 0.03$	$r = 0.7 \pm 0.2$ $p = 0.01$	$r = 0.5 \pm 0.3$ $p = 0.07$
v_{elo}				$r = 0.9 \pm 0.2$ $p = 2 \cdot 10^{-4}$	

Table S4 Correlations between parameters characterizing T4P density and dynamics under different stress conditions. Some scatter plots are shown in Fig. 7. Pearson's correlation coefficient r. p-values were obtained from 2-tailed test for the hypothesis that r = 0. r_p: rate of T4P production, N_p: number of T4P per cell, v_{elo}: T4P elongation velocity, v_{ret}: T4P retraction velocity, $\tau_p = l_p/v_{elo} + l_p/v_{ret}$: T4P lifetime.

Data Sheet 2 Log_2 fold changes of mRNA levels of genes involved in T4P biogenesis relative to DMSO control.

Data Sheet 3 Functional enrichment analysis.

Movie S1 Typical movie of T4P dynamics. Scale bar: 5 µm. $\Delta t = 0.05$ s. Movie runs in real time.

Movie S2 Typical movie of T4P dynamics under azithromycin treatment. Left: control, right: azithromycin treated cells. $\Delta t = 0.05$ s. Movie runs at 3 x speed.

Movie S3 Typical movie of T4P dynamics under ceftriaxone treatment. Left: control, right: azithromycin treated cells. $\Delta t = 0.05$ s. Movie runs at 3 x speed.

Movie S4 Typical movie of T4P dynamics under 2mM H_2O_2 treatment. Left: control, right: azithromycin treated cells. $\Delta t = 0.05$ s. Movie runs at 3 x speed.

Movie S4 Typical movies of T4P dynamics at different pH. Left: pH 5.5, center: pH 6.3 right: pH 7.0. $\Delta t = 0.05$ s. Movie runs at 3 x speed.

1. Zollner R*, et al.* (2019) Type IV Pilin Post-Translational Modifications Modulate Material Properties of Bacterial Colonies. *Biophys J* 116(5):938-947.
2. Aas FE*, et al.* (2007) Substitutions in the N-terminal alpha helical spine of Neisseria gonorrhoeae pilin affect type IV pilus assembly, dynamics and associated functions. *Mol Microbiol* 63(1):69-85.
3. Cronenberg T, Hennes M, Wielert I, & Maier B (2021) Antibiotics modulate attractive interactions in bacterial colonies affecting survivability under combined treatment. *Plos Pathog* 17(2).
